# Editorial: Global excellence in cellular neuropathology: Ukraine

**DOI:** 10.3389/fncel.2023.1354398

**Published:** 2024-01-08

**Authors:** Andrii Cherninskyi, Dirk M. Hermann, Elena Lukyanetz, Oleg Krishtal

**Affiliations:** ^1^Department of Cellular Membranology, Bogomoletz Institute of Physiology, Kyiv, Ukraine; ^2^Department of Neurology, University Hospital Essen, University of Duisburg-Essen, Essen, Germany; ^3^Department of Biophysics of Ion Channels, Bogomoletz Institute of Physiology, Kyiv, Ukraine

**Keywords:** hippocampus, epilepsy, blood-brain barrier, Alzheimer's disease, aging, nociception, cerebral ischemia, hypoxia

Cellular neuroscience is crucial for modern biology and medicine as it focuses on understanding the fundamental building blocks of the nervous system — the cells. Insights from cellular neuroscience contribute to our understanding of neurological disorders, brain development, and the mechanisms underlying learning and memory.

This Research Topic aims to show the diversity of modern research in the field of cellular neuroscience focusing on neuropathologies that are conducted in Ukrainian institutions.

Neuroscience in Ukraine has a rich history, dating back to the establishment of St. Volodymyr University (now Taras Shevchenko National University of Kyiv) in 1834. Professor Oleksandr Valter (1818–1889), the head of the university's anatomical theater, made a significant contribution in 1842 by discovering the vasoconstrictor action of sympathetic nerves. Another notable figure from the university is Professor Volodymyr Betz (1834–1894), a globally recognized neurobiologist, anatomist, and histologist (Kushchayev et al., 2012). He is famous for discovering the giant pyramidal neurons of the primary motor cortex, later named Betz cells. Professor Vasyl Chagovets (1873–1941), also from Kyiv University, developed the electrolytic theory of biopotentials. His student, Volodymyr Pravdich-Neminsky (1879–1952), was the first to record electrophysiological potentials from the surface of the intact skull and demonstrated their origin from the brain (Neminsky, 1913). Although he initially termed these waves “electrocerebrogram,” Hans Berger later proposed the term “electroencephalogram.”

Presently, neuroscience research is conducted in over 30 institutions, with Bogomoletz Institute of Physiology taking a leading role. The Institute's alumni contribute significantly to research institutions worldwide. The development of neuroscience at the Institute is closely tied to Danylo Vorontsov (1886–1965) and his student Platon Kostyuk (1924–2010). In 1975, the world witnessed the first intracellular perfusion (Kostyuk et al., 1975), which allowed the replacement of the cell cytoplasm by an artificial environment, opening new avenues in the study of nerve cell physiology and biophysics. Platon Kostuyk's students and colleagues achieved several groundbreaking milestones, including the discovery of “the receptor for protons” (later named acid-sensing ion channels or ASICs) (Krishtal and Pidoplichko, 1980), the first demonstration of low-threshold calcium currents, which flow through T-channels (Kostyuk et al., 1981), the existence of ionotropic purinoreceptors (P2X) in sensory neurons (Krishtal et al., 1983), and the first recording of the electrical activity of a single nicotinic acetylcholine receptor (Derkach et al., 1987), among others.

## In this Research Topic

The hippocampus stands out as one of the most extensively studied brain structures, with numerous papers dedicated to unraveling the properties of its neuronal networks. The study of Shkryl delves into the intricate spatiotemporal characteristics of calcium transients within cultured hippocampal pyramidal neurons. Employing two-dimensional fluorescence microscopy and the ratiometric dye Fura-2, the research unveils depolarization-induced Ca^2+^ transients exhibiting an asynchronous delayed rise in free Ca^2+^ concentration. Notably, diverse cellular regions display distinct resting calcium levels, with the cell nucleus exhibiting significantly lower concentrations compared to the soma, sub-membrane, and dendritic tree. The paper also sheds light on the different levels of involvement of ryanodine receptors in intracellular Ca^2+^ signaling across various cellular regions. In another paper, Shypshyna et al. investigate the impact of insulin on paired-pulse plasticity at glutamatergic synapses in hippocampal neurons during hyperinsulinemia, often associated with diabetes mellitus. Disruptions in insulin receptor signaling can lead to cognitive disorders linked to impaired synaptic plasticity. Results show that under normoinsulinemia, insulin enhances paired-pulse facilitation by stimulating glutamate release. Conversely, under hypoinsulinemia, insulin exhibits a limited impact on such facilitation parameters, suggesting potential insulin resistance. However, insulin's effect on neurons with paired-pulse depression indicates its capacity to restore normoinsulinemia-like conditions, including the restoration of glutamate release probability to control levels in their synapses. Pendeliuk and Melnick studied the excitatory synchronization of rat hippocampal interneurons (INs) during network activation in an *in vitro* setting. The complex nature of neural tissue has made understanding the mechanisms of INs' electrical activity synchronization challenging. Employing paired patch-clamp recordings in a simplified culture model with intact glutamate transmission, the research elevated network activity using field electric stimulation, akin to afferent processing *in situ*. The obtained data underscores that IN synchronization is primarily initiated and dominated by glutamatergic mechanisms, orchestrating various excitatory means within the neural system to support the process on a wholesale scale.

The research by Savotchenko et al. explores the pivotal role of thrombin in the initiation of early-onset seizures, particularly in the context of blood-brain barrier dysfunction observed in temporal lobe epilepsy. The study replicates the effects of blood-brain barrier disruption effects *in vitro*, examining the impact of modified blood plasma artificial cerebrospinal fluid on hippocampal neuron excitability and the role of thrombin in seizure susceptibility.

A set of papers is dedicated to the Alzheimer's disease (AD). The paper of Hanzha et al. investigates the impact of cerium dioxide nanoparticles (CNPs) on the viability of hippocampal neurons in the modeling of this disease. CNPs, known for their low toxicity and specific redox, antiradical properties, present exciting possibilities for biomedical applications, particularly in neurodegenerative diseases like AD. The results suggest that CNPs in the cultural media significantly reduce the number of dead hippocampal neurons in the presence of Aβ, emphasizing their neuroprotective properties, which may hold promise for the development of new treatments for AD. Yavorsky et al. investigated the influence of amyloid beta (Aβ) on impulse spiking in isolated CA1 hippocampal neurons, addressing one of the AD hallmarks. Findings reveal that Aβ1–42 influences AP generation differently across hippocampal neurons, with a shared effect of enhancing firing responses within a minute of Aβ1–42 application. Overall, the findings imply that prolonged exposure to Aβ1–42 in the cellular environment may lead to neuronal dysfunction due to a sustained increase in AP firing and a predisposition to this process. Amyloidosis was also the subject of the paper by Sokolik and Berchenko. The authors explored the cumulative effect of the action of miR-101 and curcumin encapsulated in a single liposome on a cellular model of AD and found it to be cumulative, which may be used for the development of more effective AD treatment.

Another topic of this Research Topic is dedicated to the aging of the nervous system. The study by Labunets et al. investigates the reactions of various brain cell types to the neurotoxin cuprizone and melatonin treatment in young and aging mice, considering the age-associated changes in inflammatory cells and their potential impact on the response to humoral/endocrine factors like melatonin. The research aims to evaluate changes in brain macrophages, astrocytes, T-cells, neural stem cells, and neurons in cuprizone-treated mice of different ages and assess the effects of exogenous melatonin. The research of Kyryk et al. explores age-related ultrastructural changes in 3D spheroids formed by adipose-derived multipotent mesenchymal stromal cells (ADSCs) from ovariectomized mice, considering the implications for cell therapy, especially in treating nervous system diseases. Their findings hold significance for potential therapeutic applications of ADSCs in treating diseases of the nervous system.

Nociception is also a big topic in Ukrainian neuroscience. Tissue acidification is known to activate primary nociceptors, leading to pain sensation, due to the activation of ASICs. According to Tkachenko et al., diminazene, a well-known antagonist of ASICs, does not suppress pH-induced activation of mechano-insensitive C-fibers. These results suggest that the excitation of afferent nerve terminals induced by mild acidification primarily involves Na^+^/H^+^ exchangers rather than ASICs. Another nociceptive “player” is the TRPV1 channel which is responsible for the sensation of heat. Petrushenko et al. studied the interplay between them and P2 receptors. The research investigates the dynamics of calcium transients in dorsal root ganglion neurons associated with TRPV1 channel desensitization and the impact of P2 receptor activation on this process. Anesthesia is a way to limit the sensation of pain. General anesthesia, commonly used during surgery, is known to transiently impair gastrointestinal motility, leading to postoperative ileus, which is a multifactorial condition characterized by neuromuscular failure, primarily affecting the small intestine. Despite its prevalence, there is a limited understanding of the underlying mechanisms, resulting in few effective medication options. The paper of Zholos et al. aims to provide a comprehensive overview of TRP channels, calcium signaling, and cholinergic mechanisms in the gut, detailing how general anesthetics negatively impact these processes.

Cerebral ischemia, often associated with circulatory disorders, leads to oxygen-glucose deficiency, disrupting essential pathways of cellular metabolism and causing damage to brain cells. Mini-review by Lushnikova et al. explores the role of the mechanistic target of rapamycin (mTOR) and α-ketoglutarate signaling in maintaining cellular homeostasis in the brain under ischemic conditions. Another review by Belenichev et al. explores the involvement of heat shock proteins HSP70 in endogenous neuroprotection mechanisms, emphasizing their potential as therapeutic targets. HSP70 functions as intracellular chaperones critical for maintaining cellular proteostasis under various stress conditions. Focusing on cerebral ischemia, the review outlines HSP70's role in preventing mitochondrial dysfunction, apoptosis activation, estrogen receptor desensitization, oxidative/nitrosative stress reduction, and morpho-functional changes in brain cells suggesting the development of neuroprotective agents modulating HSP70 and HIF-1a gene expression.

The impact of hypoxia on retinal ganglion cells and their neurotransmission pathways is the focus of two papers by Dumanska and Veselovsky shedding light on crucial aspects of oxygen deficiency in ocular contexts. The authors focused on the mechanisms underlying hypoxia-induced long-term potentiation of NMDA neurotransmission in the visual retinocollicular pathway and the immediate effects of acute hypoxia on high-voltage-activated calcium currents in cultured retinal ganglion cells. Together, these studies contribute to the understanding of hypoxia-induced alterations in retinal ganglion cells, paving the way for potential interventions in ocular diseases associated with oxygen deficiency.

The study of Lisakovska et al. investigated the intricate interplay between the brain's vitamin D3 auto/paracrine system and glucocorticoid-induced neurotoxicity, aiming to elucidate the relationship between D3 status and the multifaceted disturbances associated with prolonged glucocorticoid therapy. The results highlight the importance of considering vitamin D3 supplementation in mitigating the adverse effects of such therapy on the central nervous system.

Maternal antibiotic administration (MAA) during pregnancy is a common therapeutic intervention, yet its potential impact on the cognitive and neural development of offspring remains poorly understood. Using behavioral, histological, and electron microscopy analyses, Shepilov et al. demonstrated the potential pathological consequences of MAA during specific gestational windows on cognitive behavior and early brain development in the offspring of mice. The study emphasizes the importance of considering the timing of antibiotic exposure during pregnancy in understanding its long-term effects on offspring neurodevelopment.

Finally, the paper by Storozhuk explores the therapeutic potential of cannabidiol (CBD) in treating neurological diseases, emphasizing its neuroprotective, anti-epileptic, anti-inflammatory, anxiolytic, and anti-cancer properties. CBD has gained attention for its clinical use in epilepsy treatment. However, legal restrictions on Cannabis-derived CBD in many countries pose challenges. The study discusses the potential of non-cannabis plants, particularly flax (*Linum usitatissimum*), as a natural CBD source.

Despite the ongoing Russian aggression against Ukraine, the response to the call for papers for this Research Topic exceeded the initial expectations of the editorial team: 19 papers, involving 64 co-authors from Bogomoletz Institute of Physiology, other Ukrainian research institutions, and collaborative institutions worldwide. The number of accepted papers is even greater because some of the submitted manuscripts were transferred to other sections or journals. On behalf of all the authors of this Research Topic, we would like to express our gratitude to Frontiers Media S.A. for supporting Ukrainian science during these challenging times.

## Author contributions

AC: Writing—original draft. DH: Project administration, Writing—review & editing. EL: Writing—review & editing. OK: Project administration, Writing—review & editing.
